# Investigating the burden of disease dimensions (time-dependent, developmental, physical, social and emotional) among family caregivers with COVID-19 patients in Iran

**DOI:** 10.1186/s12875-022-01772-1

**Published:** 2022-06-30

**Authors:** Hojjat Sheikhbardsiri, Asghar Tavan, Parya Jangipour Afshar, Sahar Salahi, Majid Heidari-Jamebozorgi

**Affiliations:** 1 Student Research Committee, Kerman University of Medical Sciences, Kerman, Iran; 2 Health in Disasters and Emergencies Research Center, Institute for Futures Studies in Health, Kerman University of Medical Sciences, Kerman, Iran; 3 Department of Biostatistics and Epidemiology, Faculty of Public Health, Kerman University of Medical Sciences, Kerman, Iran; 4 Department of Nursing, Yasooj Branch, Islamic Azad University, Yasooj, Iran; 5 Department of Public Health, Sirjan School of Medical Sciences, Sirjan, Iran

**Keywords:** Burden, Family caregiver, COVID-19, Patients, Relevant factors

## Abstract

**Background:**

The caregivers of patients with covid-19 face constant responsibilities such as providing personal, health, and social care to family, which can be physically, and emotionally exhausting resulting in a considerable stress burden. Therefore, given the importance of the subject, this study aimed to investigate the burden of disease dimensions (time-dependent, developmental, physical, social and emotional) among family caregivers with covid-19 patients in Iran.

**Methods:**

This cross-sectional study was conducted one year after the onset of the Covid-19 outbreak in Iran. Family caregivers of Covid-19 patients discharged from the hospitals in Kerman city, Iran, were chosen by simple randomization (*n* = 1500). Data were collected utilizing a demographic characteristics inventory created by the researcher as well as the Novak and Guest Caregiver Burden Inventory. Descriptive statistics such as mean and standard deviations, frequency, and percentages and analytical statistics such as Kolmogorov–Smirnov, T-test, ANOVA, and Multivariate Linear Regression were used for data analysis using the 20, SPSS Inc., Chicago, IL Software at the level of *P* < 0.05.

**Results:**

The results demonstrated that the mean score of family caregiver burden was 2.61±0.6 and the severity of this burden was in a moderate range. The finding showed a statistical difference was seen between the family caregiver burden mean score of participants in terms of gender, duration of treatment, age and employed status. The multivariable linear regression model showed demographic variables of caregivers included (female, married, employed, elderly, low income and poor education) had a beneficial influence on family caregiver burden.

**Conclusion:**

The findings of this study can increase the awareness of health managers, about the level of burden of disease among family caregivers from the covid-19 patients and can help to provide economic, social and psychological support programs for improvement and reducing the burden of disease of caregivers during the covid-19 outbreaks.

## Background

The coronavirus disease 2019 (Covid-19) is a significant global pandemic pathogen that was first identified in Dec 2019 in Wuhan, China., and it quickly developed into a serious public health challenge with a strong likelihood of transmission [1]. Iran (the Islamic Republic of) is one of the most affected countries by COVID-19 and the first confirmed cases of COVID-19 in Iran were reported on February 19, 2020 [2–4].Since then, COVID-19 has spread rapidly across the country. Until 3 January 2020 to 29 April 2022, the Iranian Ministry of Health and Medical Education has reported 7,219,433 confirmed COVID-19 cases and 141,041 COVID-19-related deaths in Iran [5].Kerman Province, located in the southeast of the country, is the largest province of Iran, with a population of about 3 million people. Kerman province is a kind of industrial, cultural, political, academic-scientific, religious and other indicators among the provinces of the southeastern region of the Iran country [6]. The first two cases of COVID-19 in Kerman were identified in early March 2020 [7]. During the following months, COVID-19-related hospitalized cases increased more to 40,765 and COVID-19-related deaths reached 5,633 in the province [5].

The abrupt emergence of the disease can have a significant impact on someone’s mental well-being lth and restrictive policies such as quarantine, isolation, and social distancing, have an impact on the psychological well-being of people as well as emotional responses to the pandemic [3, 8, 9]. Covid-19 disease and its consequences inflict financial concerns and diminish the psychological health of people [10, 11]. The coronavirus disease has the potential to impair a patient's family and social interactions, and it not only affects patients but also caregivers [12, 13]. The possibly devastating consequences of the pandemic, as well as the limits imposed by disease control strategies, predisposed the community to substantial mental health difficulties [14]. many worries often emerge in the pandemic disasters, such as the constant need for self-protection, changes in the regular daily schedule, unexpected suspension of activities outside the home, and the wearing of masks that make it difficult to identify human faces [15]. Furthermore, when the number of individuals living together in the same place increases problems with social relations and emotionally charged reactions occur most frequently. These incidences may lead to the sudden onset of psychological symptoms in sensitive patients [16, 17].

Caregivers are persons who are mostly engaged in a patient’s care, adaptation and disease management during the treatment and recovery of illness [18]. Family caregivers provide a critical contribution to the medical and mental treatment of vulnerable patients [19]. Caregivers face constant responsibilities, such as providing personal, health, and social care to family, which can be physically and emotionally exhausting, resulting in a considerable stress burden [20]. In comparison to other caregivers, the caregivers of patients with covid-19 experience more excessive obstacles due to limited available training and resources, as well as a lack of information and standard care recommendations relating to this novel disease [18, 19]. Most family caregivers in addition to the caregiving role have other responsibilities such as their occupation accountability, housework childcare and regarding schools closing have responsibilities towards their children’s learning during the Covid-19 pandemic. They must also attempt to keep themselves and other household individuals against viral transmission, which is more complex to handle than caring for someone with other diseases or in other circumstances [20, 21]. The process of transition from a healthcare center to home care places a substantial burden on the family [22].

There is an objective and a subjective aspect to a patient's care; Objective care can be defined as the amount of time and cost spent on caring, considering economic, familial, and social costs. Subjective care burden referred to the caregiver's understanding of the care burden that encompasses psychological, emotional, and behavioural issues induced by the disease [18]. Studies suggest that home-based caregiving affects the care provider’s quality of life and satisfaction. Therefore, the burden of disease assessment on the patient's family or caregivers is essential to prepare facilities and support them [23]. However, to the best of our knowledge, no detailed study on the burden of disease among family caregivers with Covid-19 patients has been conducted to date. This study aimed to investigate the burden of disease among family caregivers with Covid-19 patients in southeast Iran. This study suggests to healthcare managers, implement suitable plans to assist caregivers and those activities are taken to decrease the burden of disease of caregivers of Covid-19 patients.

## Methods

### Design

This cross-sectional study was performed in 2021 in hospitals in Kerman city of Iran, affected by Covid-19 in March 2020.

### Participants and setting

The target population of this study was family caregivers of Covid-19 hospitalized and non-hospitalized patients. From February 2021 to April 2021 by using a census method the sample size included 1500 family caregivers of Covid-19 patients discharged from the three educational hospitals (Afzalipour, Shafa and Shahid Bahonar) supervised by Kerman University of Medical Sciences. Afzalipour Hospital was the main place for the admission of suspected and confirmed Covid-19 patients and is one of the largest hospitals in Iran. This Hospital is a 700-bed therapeutic-educational hospital with an occupancy rate of 75 percent. Afzalpour Hospital included five intensive care units (ICUs) with 52 active beds (Covid- 19 ICU, general ICU, surgical ICU, poisoning ICU and neonatal and pediatric ICU). Shafa Hospital is a 615-bed therapeutic-educational hospital with an occupancy rate of 54 percent and included five ICUs with 30 active beds. Shahid Bahonar Hospital is a 370-bed therapeutic-educational hospital with 51,000 admissions annually and an occupancy rate of 67 percent and included four ICUs with 48 active beds.

Due to the Covid-19 outbreak, data were collected through online questionnaires to minimize the Covid-19 transmission risk between researchers and respondents. We first extracted patients' contact information from the information system of these hospitals and then the survey was shared electronically using an online service system (kmu.ac.ir/fa/formadd/38570); the link to the survey was disseminated via social media (Telegram, WhatsApp, and Instagram). Names of participants or other personal characteristics were not included in the questionnaires to maintain confidentiality. In total, 1500 questionnaires were distributed; 988 questionnaires were returned out of which 34 incomplete questionnaires were excluded. An overall response rate was 65.4% for inclusion in the analyses.

### Inclusion and exclusion criteria

Inclusion requirements consist of a lack of mental disorder, the ability to read and write, being at least 18 years old, and a tendency to take part in the research. Exclusion consideration was disagreement for participation and missing questionnaires.

### Data collection

Data was collected using a demographic variables questionnaire created by the researcher and Novak and Guest Caregiver Burden Inventory (CBI) to investigate family caregiver burden (FCB) among family caregiver with covid-19 patients in Iran. Demographic characteristics included age, gender, education level, treatment length, marital status, employment status, caregiver income and relationship to the patient. CBI is comprised of 24 questions divided into five categories: time-dependent burden (Items 1 to 5), developmental burden (Items 6 to 10), physical burden (Items 11 to 14), social burden (Items 15 to19), and emotional burden (Items 20 to 24). All of the questions were rated on a five-point Likert measure (never = 1, almost always = 5), having higher scores indicating greater burden [24]. Five-point responses were scored from 1 to 5 and the mean scores were calculated. Therefore, the base of total mean scores the low burden is defined as 1 to 2.33 points, moderate is 2.34 to 3.67 points, and severe is 3.68 to 5 points.

Briefly, time dependence burden evaluates stress caused by the restriction of one's personal time due to time demands of caregiving whereas developmental burden describes a sense of failure in development concerning their peers. Physical burden refers to the impact on caregivers' physical health, strength, and energy while social burden implies feelings of role conflict concerning one's job or family. Finally, emotional burden represents negative feelings, embarrassment or feelings of shame caused by the patient [24].

Ten professors of the Kerman University of Medical Sciences confirmed the questionnaire content validity. For reliability, preliminary research was performed over the scale of the burden of disease. The questionnaire was given to thirty family caregivers who were not included in the research. The reliability was determined using Cronbach's alpha coefficient, which was 83 percent.

### Data analysis

Descriptive statistics such as mean and standard deviations, frequency, and percentages were used to analyze the data and The Kolmogorov–Smirnov test was implemented to determine if the data were distributed normally. The T-test and ANOVA tests were performed to compare mean scores of the burden of disease based on participants' demographic characteristics. The multivariate regression was undertaken to investigate the effect of demographic factors on FCB using the 20, SPSS Inc., Chicago, IL Software at the level of *P* < 0.05.

## Results

Of all the 954 participants, 314 were Male (32.9%) and 640 were female (67.1%). Of 954 caregivers, 699 (73.3%) were married and 255 (26.7%) were single. The level of education of the majority of caregivers 335 (35.1%) was a bachelor's academic degree. Other participants' demographic characteristics are displayed in Table 1.

**Table 1:** Demographic characteristics (*N* = 954)

Variables	Type	Number	Percentage
**Gender**	Male	314	32.9
	Female	640	67.1
**Age**	20–30	157	16.5
	31–40	570	59.7
	Above 40	227	23.8
**Marital status**	single	255	26.7
	Married	699	73.3
**Education Level**	Diploma and sub diploma degree	165	17.3
	Associate degree	192	20.1
	Bachelor academic degree	335	35.1
	Master academic degree	262	27.5
**Employment status**	Employed	548	57.4
	Unemployed	406	42.6
**Duration of treatment**	Low 7 day	104	10.9
	7–14	354	37.1
	Up 14 day	496	51.9
**Relationship to The Patient**	Father	73	7.3
	Mother	361	37.8
	Sister	300	31.4
	Brother	94	9.8
	Grandmother	98	10.2
	Grandfather	28	2.93
**Caregiver income**	≤ 5 million tomans per month	605	63.4
	5–10 million toman per month	247	25.8
	≥ 10 million tomans per month	102	10.6

The results showed that the mean and standard deviation of FCB was 2.61±0.6 and the severity of this burden was at a moderate level. The results of our study showed among FCB domains the mean score of the time-dependent caregiver burden domain with a mean score of 3.00 ± 0.8 was at a burden high level and the Physical caregiver burden domain with a mean score of 2.26 ± 0.9 was at a burden Lowe level, shown in Table 2.

**Table 2:** The Mean score of FCB domain’s

**Variables**	**Mean ± SD**
**Time-dependent burden**	3.00±0.8
**Developmental burden**	2.48±0.8
**Physical burden**	2.26±0.9
**Social burden**	2.44±0.9
**Emotional burden**	2.80±0.9
**Total mane score of Caregiver burden**	2.61±0.6

The T-test revealed a statistically significant difference in the mean scores of FCB (*P* < 0.001) in terms of gender. The results base on mean ranks demonstrated that females had more care burden in comparison with males, as shown in Table 3. The T-test revealed a statistically significant difference in the mean score of FCB in terms of participants' employment status (*P* < 0.001), the results base on mean ranks showed the employed caregiver 'had more care burden in comparison with the unemployed caregiver, shown in Table 3.

**Table 3:** T-test for comparison of FCB dimensions mean score with gender and employment status

**Variables**	**Gender**	**Mean ± SD**	***p*-value**
Time-Dependent Burden	Female	3.08±0.5	< 0.001*
	Male	2.96±0.9	
Developmental Burden	Female	2.75±0.8	< 0.001*
	Male	2.34±0.8	
Physical Burden	Female	2.42±0.8	< 0.001*
	Male	2.18±0.9	
Social Burden	Female	2.83±0.8	< 0.001*
	Male	2.24±0.9	
Emotional Burden	Female	2.93±0.8	< 0.001*
	Male	2.74±0.9	
Total Mane Score of FCB	Female	2.81±0.4	< 0.001*
	Male	2.50±0.6	
**Variables**	**Employment Status**	**Mean ± SD**	***P*-Value**
Time-Dependent Burden	Unemployed	2.93±0.7	< 0.001*
	Employed	3.10±0.9	
Developmental Burden	Unemployed	2.38±0.8	< 0.001*
	Employed	2.62±0.8	
Physical Burden	Unemployed	2.11±0.8	< 0.001*
	Employed	2.46±0.9	
Physical Burden	Unemployed	2.32±0.9	< 0.001*
	Employed	2.59±0.8	
Emotional Burden	Unemployed	2.63±0.9	< 0.001*
	Employed	3.02±0.8	
Total Mane Score of FCB	Unemployed	2.49±0.6	< 0.001*
	Employed	2.77±0.5	

The ANOVA test revealed a statistically significant difference in the mean score of FCB in terms of duration of treatment (*P* < 0.00). Therefore, the base of mean ranks the caregivers with the duration of treatment Up 14 Days for patients are more care burden. The ANOVA test showed a statistically significant difference in the mean score of FCB in terms of age (*P* < 0.00). According to the mean values of the ranks, the rate of care burden is higher in caregivers above 40, as shown in Table- 4. There was no statistically significant difference between other demographic characteristics of participants and dimensions of FCB.

**Table 4:** ANOVA test for comparison of FCB dimensions mean score with the duration of treatment and age

**Variables**	**Duration of Treatment**	**Mean ±SD**	***P*-Value**
Time-Dependent Burden	Low 7 Day	2.70±0.5	<0.001*
	14-Jul	3.01±0.9	
	Up 14 Day	3.15±0.6	
Developmental Burden	Low 7 Day	2.36±1.0	<0.001*
	14-Jul	2.46±0.8	
	Up 14 Day	2.52±0.8	
Physical Burden	Low 7 Day	2.23±0.8	<0.001*
	14-Jul	2.26±1.3	
	Up 14 Day	2.30±1.0	
Social Burden	Low 7 Day	2.36±0.8	<0.001*
	14-Jul	2.42±1.0	
	Up 14 Day	2.44±0.7	
Emotional Burden	Low 7 Day	2.30±0.6	<0.001*
	14-Jul	2.71±0.8	
	Up 14 Day	2.87±0.9	
Total Mane Score of FCB	Low 7 Day	2.45±0.6	<0.001*
	14-Jul	2.59±0.6	
	Up 14 Day	2.62±0.6	
**Variables**	**Age**	**Mean ±SD**	***P*-Value**
Time-Dependent Burden	20-30	2.82±0.6	<0.001*
	31-40	2.94±0.8	
	Above 40	3.09±0.8	
Developmental Burden	20-30	2.37±0.9	<0.001*
	31-40	2.44±0.8	
	Above 40	2.53±0.8	
Physical Burden	20-30	2.17±0.7	<0.001*
	31-40	2.33±0.9	
	Above 40	2.48±0.7	
Social Burden	20-30	2.11±0.7	<0.001*
	31-40	2.53±0.9	
	Above 40	2.57±0.7	
Emotional Burden	20-30	2.51±0.8	<0.001*
	31-40	2.84±0.9	
	Above 40	3.04±0.8	
Total Mane Score of FCB	20-30	2.50±0.5	<0.001*
	31-40	2.63±0.6	
	Above 40	2.67±0.6	

The multivariable linear regression model showed demographic variables of caregivers included (female, married, employed, elderly, low income and poor education) had a beneficial influence on FCB. In addition, the multivariable linear regression model demonstrated that increasing the duration of treatment had a positive impact on caregivers, as Shown in Table 5.

**Table 5:** Multiple regression for evaluating the effect demographic variables on FCB

**Variable**	**Group**	**Coefficient**	**Std.Error**	***p*-value**
Age	20–30	reference		
	31–40	0.023	0.032	0.689
	Above 40	0.105	0.048	0.012*
Sex	Male	reference		
	Female	0.147	0.019	< 0.001*
Education Level	Diploma and sub diploma	0.253	0.043	< 0.001*
	Associate degree	0.094	0.048	0.055
	Bachelor	0.026	0.065	0.689
	Master	reference		
Duration of Treatment	Low 7 day	reference		
	7–14	0.094	0.020	< 0.001*
	Up 14 day	0.130	0.029	< 0.001*
Marital Status	single	reference		
	Married	0.135	0.046	< 0.001*
Employment Status	Unemployed	reference		
	Employed	0.232	0.041	< 0.001*
Relationship to the Patient	Father	0.090	0.021	< 0.001*
	Mother	0.180	0.058	< 0.001*
	Sister	0.112	0.038	< 0.001*
	Brother	0.075	0.066	0.263
	Grandmother	0.043	0.119	0.442
	Grandfather	reference		
Caregiver Income	≤ 5 million tomans per month	0.125	0.020	< 0.001*
	5–10 million toman per month	0.115	0.050	< 0.001*
	≥ 10 million tomans per month	reference		

## Discussion

Family caregivers experience various challenges because of restricted access to alternative caregiving resources and worries for their loved ones' physical and mental well-being [19]. The present study was designed to investigate the caregiver burden among caregivers of Covid-19 patients admitted and discharged from the three treatment-educational hospitals as the main treatment centers for Covid-19.

The findings of the study demonstrated that the mean score of care burden in participants was at a moderate level. This result was consistent with the study of [18] in Iran and the study of [25] In the United State but inconsistent with the study of [26] in Japan and the study of turkey [27]. The reason for this difference can include differences in the type of study, the type of illness of the individual in the family, the underlying disease of the patient in the family, differences in the instrument, the study setting and the difference in the educational system of countries at the community level. Radio and Television of Iran by creating appropriate training channels for caregivers with Covid-19 inpatients and outpatients provides the necessary training during the day by various experts in various fields of medical sciences, especially clinical and general psychologists that can reduce the FCB.

According to the findings of this study, among the five domains of FCB, time-dependent burden received the highest score among caregivers compared to the other domains. A search through available databases did not reveal a study examining the burden of disease in family caregivers of Covid-19 patients, although there were studies on the burden of disease of caregivers in other diseases including studies [28, 29]. This domain represents the length of time caregivers devote each day caring for their patients. It seems that this result relies on the significant dependency of Covid-19 patients on taking drugs and their requirements for medical attention. Furthermore, owing to the scarcity of home care facilities in developing countries such as Iran, patients must seek treatment at clinics or public health centers. As a result, caregivers expend a significant amount of time.

The findings of the present research demonstrated that among five domains of FCB, physical caregiver burden in comparison to other aspects scored the lowest level. This result was inconsistent with the studies of [30–32]. These studies revealed that caregivers are exposed to a considerable degree of physical and mental pressure when caring for a patient. The reason for the discrepancy in this study could be that dialysis patients need several years of care, while covid-19 patients care for much less time. In addition, Iranian families at our place of study are at a high level in terms of emotionality and friendliness. Due to the nature of the disease and the high mortality rate, patient caregivers make every effort to care for the patient and complain less about the physical burden.

The results showed a statistically significant difference in the mean scores of FCB in terms of gender. The results based on mean ranks showed that females had more care burden in comparison with males. This result was consistent with the studies of [18, 19] in Iran country. This concern is directly associated with the Iranian socio-cultural context, in which the responsibility of child care, the older member, members with disabilities and the ill person is frequently integrated with homemakers and regarded as a part of housekeeping. Another reason for discussing this result is that in our study the majority of the family caregivers of Covid-19 patients were women. A study of Muslim women in Turkey found that women were less likely to become pregnant during the Covid-19 pandemic. This could be due to the extra role of women and the greater workload in the family during this period [33].

The finding revealed a statistically significant difference in the mean score of FCB in terms of participant’s employment status, the results based on mean ranks showed the employed caregiver had more care burden in comparison with an unemployed caregiver. This result was consistent with the studies of [19, 34, 35]. This finding is not unexpected because in Iranian culture, the patient's family is expected to offer financial assistance, and if the patient has a poor income, the caregiver will be required to spend more for patient care, putting a heavier burden on the caregiver. As a result, to offer appropriate care for Covid-19 patients, it is recommended to develop approaches to supply and provide funding assistance to caregivers.

The results of this study demonstrated a statistically significant difference in the mean score of FCB in terms of duration of treatment. Therefore, the base of mean ranks the caregivers with the duration of treatment up 14 days for patient are more care burden. This result was consistent with the studies [36, 37]. This finding is not unexpected because family caregivers are frequently the fathers or mothers, spouses and other members who have the greatest interaction with the patient during the curing process and who frequently suffer from emotional issues such as worry, depression, sadness, disappointment, anger, isolation, fear, and anxiety. Therefore, due to the unknown nature of the disease, the longer course of the Covid-19 disease, the patient's family will experience a more caring burden [17, 38].

The results of this study demonstrated a statistically significant difference in the mean score of FCB in terms of age. According to the mean values of the ranks, the rate of care burden is higher in caregivers above 40. This result was consistent with the studies [39, 40]. Caregiving roles and responsibilities have increased for all family caregivers during the pandemic, older caregiver appears to be more distressed by these shifts in care for their patient’s Covid-19. On the other hand, most people over the age of 40 usually have an underlying disease and need to take care of themselves, caring for another patient may sometimes be difficult, and impossible for them therefore these caregivers may experience more care burden.

### Limitation

The first possible limitations of the current study were a lack of cooperation of the participants and unwillingness to respond to the items truly due to embarrassment and worry of disclosing information. These restrictions were somewhat solved by effectively interacting with the participants and informing them that cooperation is voluntary, that their responses will be preserved confidential, and that they can complete the form without putting their names on it. The second limitation of this study was that we only used a questionnaire to assess FCB, therefore for final approval and definitive diagnosis need to use the appropriate diagnostic tool such as the structured clinical interview. The third limitation was that the findings are not representative of the broader population of Covid-19 patients, and the findings of this research will not apply to entire Covid-19 patients in any community around the world, finally, four limitations it was that the type of covid-19 is not segregated by severity, and caring for mild patients is less burdensome than caring for severe patients.

## Conclusion

Based on the findings, it is critical to pay more consideration to the difficulties and demands of Covid-19 patients' home caregivers. Health-care managers should give patients’ relatives enough awareness and financial assistance. There is also a need to establish programs and actions to alleviate the care burden on Covid-19 patients' family caregivers. Furthermore, society's emotional environment should be kind to patients and families to develop compassion and support patient care at home. It is recommended that factors such as quality of life, perceived social support, and disturbances like depression, fear, and anxiety should be explored in further studies within this population of caregivers during the covi19 pandemic.

## Data Availability

The data sets generated during the current study are available from the corresponding author.
